# α-Melanocyte-stimulating-hormone (α-MSH) modulates human chondrocyte activation induced by proinflammatory cytokines

**DOI:** 10.1186/s12891-015-0615-1

**Published:** 2015-06-21

**Authors:** Franco Capsoni, Anna Maria Ongari, Caterina Lonati, Riccardo Accetta, Stefano Gatti, Anna Catania

**Affiliations:** Allergy, Clinical Immunology & Rheumatology Unit, Istituto Auxologico Italiano, IRCCS, University of Milan, Piazzale Brescia, 20 - 20149 Milano, Italy; Center for Preclinical Investigation, Fondazione IRCCS Ospedale Maggiore Policlinico, Mangiagalli e Regina Elena, Milan, Italy; Traumatology and First Aid Unit, Istituto Ortopedico Galeazzi, IRCCS, Milan, Italy

## Abstract

**Background:**

Alpha-melanocyte-stimulating-hormone (α-MSH) has marked anti-inflammatory potential. Proinflammatory cytokines are critical mediators of the disturbed cartilage homeostasis in osteoarthritis, inhibiting anabolic activities and increasing catabolic activities in chondrocytes. Since human chondrocytes express α-MSH receptors, we evaluated the role of the peptide in modulating chondrocyte production of pro-inflammatory cytokines, matrix metalloproteinases (MMPs), tissue inhibitors of MMPs (TIMPs), inducible nitric oxide synthase (iNOS) and nitric oxide (NO) in response to interleukin-1β (IL-1β) and tumor necrosis factor-α (TNF-α).

**Methods:**

Human articular chondrocytes were obtained from osteoarthritic joint cartilage from subjects undergoing hip routine arthroplasty procedures. The cells were cultured with or without α-MSH in the presence of IL-1β or TNF-α. Cell-free supernatants were collected and cells immediately lysed for RNA purification. Expression of cytokines, MMPs, TIMPs, iNOS was determined by Reverse Transcription Real-time Polymerase Chain Reaction and enzyme-linked immunosorbent assay. Griess reaction was used for NO quantification.

**Results:**

Gene expression and secretion of IL-6, IL-8, MMP-3, MMP-13 were significantly increased in IL-1β or TNF-α-stimulated chondrocytes; α-MSH did not modify the release of IL-6 or IL-8 while the peptide significantly reduced their gene expression on TNF-α-stimulated cells. A significant inhibition of MMP3 gene expression and secretion from IL-1β or TNFα-stimulated chondrocytes was induced by α-MSH. On the other hand, α-MSH did not modify the release of MMP-13 by cytokine-stimulated chondrocyte but significantly decreased gene expression of the molecule on TNF-α-stimulated cells. Detectable amount of TIMP-3 and TIMP-4 were present in the supernatants of resting chondrocytes and a significant increase of TIMP-3 gene expression and release was induced by α-MSH on unstimulated cells. TIMP-3 secretion and gene expression were significantly increased in IL-1β-stimulated chondrocytes and α-MSH down-regulated gene expression but not secretion of the molecule. TIMP-4 gene expression (but not secretion) was moderately induced in IL-1β-stimulated chondrocytes with a down-regulation exerted by α-MSH. IL-1β and TNF-α were potent stimuli for NO production and iNOS gene expression by chondrocytes; no inhibition was induced by α-MSH on cytokine-stimulated NO production, while the peptide significantly reduced gene expression of iNOS.

**Conclusions:**

Our results underscore a potential anti-inflammatory and chondroprotective activity exerted by α-MSH, increasing TIMP-3 gene expression and release on resting cells and down- modulating TNF-α-induced activation of human chondrocytes. However, the discrepancy between the influences exerted by α-MSH on gene expression and protein release as well as the difference in the inhibitory pattern exerted by α-MSH in TNF-α- or IL-1β-stimulated cells leave some uncertainty on the role of the peptide on chondrocyte modulation.

## Background

Osteoarthirits (OA) is a chronic rheumatic disease characterized by cartilage degradation and loss, subchondral bone remodelling, and possible synovial inflammation. The precise cause of OA is unknown. A failure of chondrocytes to maintain the balance between synthesis and degradation of the cartilage extracellular matrix is considered a significant factor in the loss of cartilage [1].

The assumed mechanisms involved in chondrocyte dysregulation and/or apoptosis include mechanical stress, age-related functional changes and altered production of pro-inflammatory cytokines, predominantly interleukin-1β (IL-1β) and tumor necrosis factor-α (TNF-α), that induce production of oxygen radicals and proteinases such as matrix metalloproteinases (MMPs) and aggrecanases [2]. These observations suggested that anti-cytokine and anti-oxydant compounds could have chondroprotective effects providing novel therapeutic opportunities for OA treatment [3, 4].

α-Melanocyte-stimulating hormone (α-MSH) is an endogenous tridecapeptide that exerts multiple effects on host cells [5]. The natural peptide and its synthetic analogs inhibit inflammatory response in experimental models of acute and chronic disorders, including inflammatory bowel diseases, allergy, adjuvant arthritis, and sepsis [6–9]. α-MSH interacts with host cells through activation of four of the five recognized melanocortin receptors (MCR), specifically MCR 1, 3, 4 and 5 [9]. The anti-inflammatory action of the peptide depends primarily on inhibition of cytokine production by target cells, although other leukocyte functions, including reactive oxygen intermediate (ROI) production, nitric oxide (NO) generation and release of proteolytic enzymes, are likewise influenced [7–9].

In spite of substantial evidence suggesting a beneficial effect of melanocortin peptides in control of numerous inflammatory disorders and the observation that human chondrocytes express MCR [10], only recently the therapeutic potential of melanocortins in OA has been explored. Yoon et al. [11], using a human chondrosarcoma cell line (HTB-94) showed that α-MSH inhibited TNF-α-induced expression of MMP-13, through a decrease in mitogen-activated protein kinase (MAPK) p38 phosphorylation and subsequent activation of nuclear factor-κB (NF-κB). In human articular chondrocytes, α-MSH decreased IL-1β and TNF-α mRNA but increased the secretion of TNF-α, IL-6 and Transforming Growth Factor-β1 (TGF-β1) [10]. In addition, in rodent chondrocytes, adrenocorticotropin (ACTH) treatment promoted development of the chondrocyte phenotype through activation of the MCR 3 [12]. Recently Kaneva et al. [13], using a human chondrocyte cell line (C-20/A4) demonstrated that melanocortin peptides strongly modulated chondrocyte function: the molecules inhibited TNF-α-induced production of pro-inflammatory cytokines whilst increasing production of the anti-inflammatory and chondroprotective cytokine interleukin-10 (IL-10); decreased gene expression of MMPs; prevented chondrocyte apoptosis by inhibiting TNF-α induced caspase-3 and -7 activation.

The main obstacle in studies on human chondrocyte biology depends on the difficulty to obtain primary articular chondrocytes as these cells lose chondrocytic phenotype when expanded and cultured in monolayer. In the present study we evaluated the capacity of primary articular human chondrocytes to produce pro-inflammatory cytokines, MMPs, tissue inhibitors of MMPs (TIMPs) and NO in response to pro-inflammatory cytokines (IL-1ß and TNF-α). In parallel experiments the effects of α-MSH in modulation of mediator production were investigated.

## Methods

### Chondrocyte isolation and culture

Samples of adult human articular cartilage were harvested from subjects undergoing hip routine arthroplasty procedures at the Istituto Ortopedico Galeazzi IRCCS, Milan. This study did not undergo ethical approval since the cartilage was collected as waste material after receiving patients signed informed consent and the approval of the Institutional Review Board (IRCCS Istituto Ortopedico Galeazzi)*.*

Minced cartilage fragments (2-3 mm^2^) were submitted to enzymatic digestion with 0.15 % collagenase type II (Worthington Biochemical Corporation, Lakewood, NJ, USA) overnight at 37 °C., as previously described [14]. Freshly isolated chondrocytes were plated for expansion at a density of 1x10^4^ cells/cm^2^ and cultured with complete medium (CM) containing Dulbecco’s Modified Eagle Medium (DMEM) supplemented with 10 % fetal bovine serum (FBS, Lonza Group Ltd, Basel, CH), 1 mM sodium pyruvate, 100 mM HEPES buffer, 100 U/mL penicillin, 100 μg/mL streptomycin, 0.29 mg/mL L-glutamine [15]. When the cells reached 80–90 % of confluence, they were detached with trypsin/EDTA (0.5 % trypsin/0.2 % EDTA and washed before performing the assays. Human chondrocytes were placed in 15 ml tissue culture tubes for 4 h with either phosphate buffered solution (PBS) (control) or α-MSH 10^−6^M. The tests employed the analogue Nle4,DPhe7-α-MSH (NDP-MSH), a non specific MC agonist that exerts similar effects relative to the natural α-MSH and is generally preferred for its greater chemical stability [16]. The cells were then cultured for 40 h in the presence or absence of 10 ng/mL human recombinant IL-1β or 10 ng/mL human recombinant TNF-α [17]. In our preliminary experiments, these culture conditions had been found to be optimal to activate human chondrocytes. Where not otherwise specified, Sigma-Aldrich (Poole, Dorset, UK) products were used. Cell-free supernatants were collected and stored at -20 °C and cells immediately lysed for RNA purification.

### Cytokine quantification by ELISA

A qualitative commercial enzyme-linked immunosorbent assay test (Multi-analyte ELISArray kit (SABiosciences - QIAGEN, Maryland, USA) has been used to simultaneously profile the level of multiple cytokines (IL-1α, IL-1ß, IL-2, IL-4, IL-6, IL-8, IL-10, IL-12, IL-17A, IFNγ, TNF-α, GM-CSF) in cell culture supernatants.

A cutoff of twice the absorbance value (read at 450 nm) of the negative control for every cytokine (A_450_) was used and the results were reported as positive (A_450_ ≥ specific cutoff) or negative (A_450_ < specific cutoff).

Protein concentration of detectable cytokines IL-6, IL-8, MMP-3, MMP-13 (Boster Biological Technology, Fremont, CA, USA), TIMP-3 and TIMP-4 (R&D Systems, Minneapolis, MN, USA) was then determined in cell-free supernatants using quantitative commercially available ELISA kits.

### Quantification of nitrites

The total NO production was measured using a commercial kit that involves the enzimatic conversion of nitrate to nitrite by the enzyme Nitrate Reductase (Enzo Life Sciences - Farmingdale, NY, USA). Briefly, 50 μl of the culture supernatant and 50 μl of Nitrate Reductase were mixed and incubated for 30 min at 37 °C in 96-well plates. One hundred microliters of the Griess reagent were added to the wells and incubated for further 10 min before reading the optical density at 540–570 nm. The nitrite concentration was calculated from a standard curve of sodium nitrate and expressed as μmole/l.

### RNA purification

Total RNA was isolated from chondrocyte lysates by anion exchange chromatography using RNeasy MinElute columns (RNeasy Mini Kit, Qiagen Inc., Hilden, Germany). Briefly, cell lysates were added (v/v) to 70 % ethanol and immediately transferred to an RNeasy column. DNase I treatment (Qiagen) was performed to remove genomic DNA contamination. After two washes in ethanol-based buffer (Buffer RPE, Qiagen), RNA were eluted in RNase-free water and then quantified by optical density measurement using Nanodrop ND-100 spectrophotometer (Nanodrop Technologies, Wilmington, DE). Each sample showed a 260/280 ratio between 1.8 and 2. RNA integrity was assessed by electrophoresis on denaturing agarose–formaldehyde gels.

### Reverse Transcription Real-time Polymerase Chain Reaction (Real-time RT-PCR) analysis

Gene expression was evaluated by Real-Time Reverse Transcription-Polymerase Chain Reaction (RT-PCR) analysis. Two micrograms of total RNA were reverse transcribed to single stranded cDNA using SuperScript VILO cDNA Synthesis Kit (Life Technologies, Foster City, CA) according to the manufacturer's instructions. Real-time PCR based on TaqMan chemistry was performed with 20 ng of retrotranscribed RNA, 1X FastStart Universal Probe Master (Roche Diagnostics GmbH, Mannheim, Germany), and predesigned 900 nM primers and 250 nM probe mix (Taqman Gene Expression Assays, Life Technologies, (assay IDs are listed in Table 1) in a final volume of 10 μl. Amplification reactions were carried out on an ABI PRISM 7900HT sequence detection system (Applied Biosystems, Life Technologies) with the following thermal profile settings: an initial step of 10 min at 95°c to activate the FastStart Taq DNA polymerase, then 50 cycles of 95 °C for 10 s and 60 °C for 30 s. Three independent PCR amplification experiments were performed for each transcript. Fluorescence intensities were converted in threshold cycles (Ct) using ABI Prism SDS 2.3 software (Life Technologies); baseline and threshold were set by automatic analysis. Relative quantification of target gene expression was calculated with the ΔCt method, using the average Ct across basal samples as calibrator for each gene. Analysis of reference genes stability, performed with the geNorm software, indicated HPRT1 and GAPDH as the most stable [18].

**Table 1:** Gene Symbols and Assay ID of the genes evaluated using Real-time RT-PCR

Gene name	Gene symbol	Assay ID
IL6	interleukin 6	Hs00174131_m1
IL8	interleukin 8	Hs00174103_m1
MMP3	matrix metalloproteinase 3	Hs00968305_m1
MMP13	matrix metalloproteinase 13	Hs00233992_m1
TIMP1	TIMP metallopeptidase inhibitor 1	Hs00171558_m1
TIMP3	TIMP metallopeptidase inhibitor 3	Hs00165949_m1
TIMP4	TIMP metallopeptidase inhibitor 4	Hs00162784_m1
ACTB	actin, beta	Hs99999903_m1
GAPDH	glyceraldehyde-3-phosphate dehydrogenase	Hs99999905_m1
RPLP0	ribosomal protein, large, P0	Hs99999902_m1
HPRT1	hypoxanthine phosphoribosyltransferase 1	Hs99999909_m1

### Statistical analysis

The data are expressed as the mean ± standard error of the mean (S.E.M.) from 4 determinations as indicated. Statistical analysis was done using Student’s *t* test for unpaired data, as appropriate. Differences were considered to be statistically significant at *P* < 0.05.

## Results

### Effect of pro-inflammatory cytokines on human chondrocytes

Of the twelve cytokines evaluated in the supernatants of IL-1β or TNF-α-stimulated chondrocytes with a qualitative ELISA test (IL-1α, IL-1ß IL-2, IL-4, IL-6, IL-8, IL-10, IL-12, IL-17α, IFNγ, TNF-α, GM-CSF), significant levels were observed only for IL-6 and IL-8 (not shown). The protein concentration of these two cytokines is shown in Table 2. Gene expression of IL-6 and IL-8 was also significantly increased in IL-1β or TNF-α-stimulated chondrocytes (Table 3). Increased levels of MMP-3 and MMP-13 in the supernatants (Table 2) as well as enhanced gene expression (Table 3) were observed in cytokine-stimulated chondrocytes, with IL-1β being the most potent stimulus.

**Table 2:** Effect of IL-1β and TNF-α on secretion of different molecules by human chondrocytes

	Stimulus		
Molecules in the Supernatants (ng/mL)	Medium	IL-1β	TNF-α
IL-6	3.0 ± 0.2	367 ± 4.9*	70 ± 3.7*
IL-8	0.0	781 ± 32*	120 ± 5.6*
MMP-3	2.0 ± 0.00	14500 ± 400*	8100 ± 50*
MMP-13	8.1 ± 0.5	71.0 ± 8.0*	35.3 ± 2.7*
TIMP-3	1.63 ± 0.12	2.71 ± 0.05°	1.23 ± 0.01 ns
TIMP-4	0.11 ± 0.01	0.12 ± 0.01 ns	0.12 ± 0.01 ns
NO (μM)	19.5 ± 3.5	102.8 ± 32.6*	95.8 ± 36.9*

Primary human chondrocytes were cultured for 40 h in medium or in the presence of 10 ng/mL IL-1ß or TNF-α. The cell-free supernatants were collected and the concentration of interleukins, MMPs, TIMPs and NO were measured. Data from 4 separate experiments (± S.E.M.) are shown

* *p* < 0.0001; °*p* < 0.002; ns: not significant vs medium

**Table 3:** Effect of IL-1ß and TNF-α on gene expression of different molecules in human chondrocytes

	Stimulus	
Relative gene expression for	IL-1β	TNF-α
IL-6	2767 ± 176*	680 ± 49*
IL-8	5090 ± 271*	1088 ± 78*
MMP-3	112 ± 10.0*	71 ± 6.5*
MMP-13	7.5 ± 2.0*	6.5 ± 0.5*
TIMP-3	3.4 ± 0.7°	1.0 ± 0.1 ns
TIMP-4	1.9 ± 0.2°	1.0 ± 0.05 ns
iNOS	1656 ± 100*	1663 ± 110*

Primary human chondrocytes were cultured for 40 h in medium or in the presence of 10 ng/mL IL-1ß or TNF-α. RNA was purifiedd and analyzed by Real-time RT-PCR. Gene expression data were calculated with the ΔCT method and using HPRT1 and GAPDH as reference genes. Relative expression was obtained comparing samples to untreated control whose gene expression was considered as baseline. Data from 4 separate experiments (± S.E.M.) are shown

* *p* < 0.0001; °*p* < 0.02; ns: not significant vs untreated controls

Low but detectable amounts of TIMP-4 (Table 2) were present in the supernatants of non-stimulated chondrocytes with no increased secretion after stimulation by IL-1β or TNF-α. TIMP-4 gene expression was moderately induced by IL-1β while there were no changes when the stimulus was TNF-α (Τable 3). TIMP-3 was also present in the supernatants of resting chondrocytes and its secretion (Table 2) as well as gene expression (Table 3) were significantly increased by IL-1β but not by TNF-α stimulation. Both IL-1β and TNF-α were potent stimuli for NO production (Table 2) and iNOS gene expression (Table 3).

### Effect of α-MSH on human chondrocytes

**Fig. 1:**
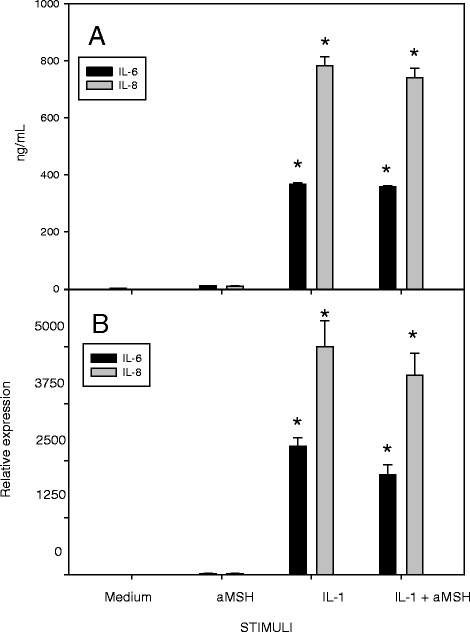
Effect of α-MSH on IL-6 and IL-8 secretion (**a**) and gene expression (**b**) in IL-1β-stimulated chondrocytes. Human primary chondrocytes were incubated for 4 h at 37 °C with 10^−6^M α-MSH and further cultured for 40 h in the presence or absence of 10 ng/mL IL-1β. IL-6 and IL-8 concentration in the supernatans (**a**) and gene expression (**b**) were then measured. Data from 4 separate experiments (± S.E.M.) are shown. *p < 0.0001 vs Medium

**Fig. 2:**
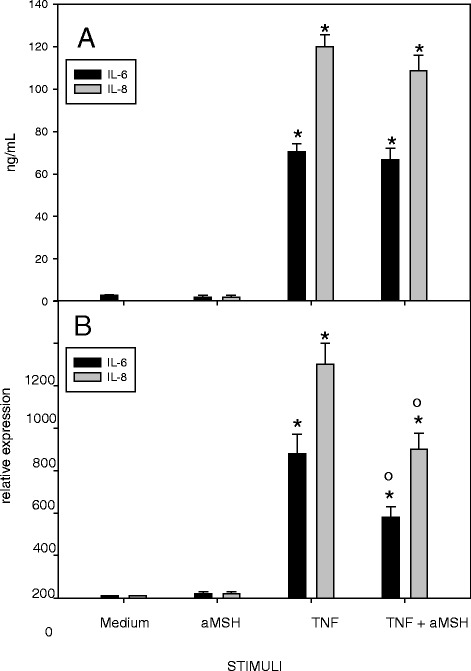
Effect of α-MSH on IL-6 and IL-8 secretion (**a**) and gene expression (**b**) in TNF-α-stimulated chondrocytes. Human primary chondrocytes were incubated for 4 h at 37 °C with 10^−6^M α-MSH and further cultured for 40 h in the presence or absence of 10 ng/mL TNF-α. IL-6 and IL-8 concentration in the supernatans (**a**) and gene expression (**b**) were then measured. Data from 4 separate experiments (± S.E.M.) are shown. *p < 0.0001 vs Medium; **°** *p* < 0.05 vs TNF-α

Pre-treatment of chondrocytes with 10^−6^M α-MSH did not modify the release of IL-6 or IL-8 by IL-1ß (Fig. 1) or TNF-α (Fig. 2) stimulated chondrocytes. On the other hand, while only slightly decreased gene expression of IL-6 and IL-8 was observed when the cells were stimulated with IL-1ß (Fig. 1), the reduction became significant when the stimulus was TNF-α (Fig. 2)

**Fig. 3:**
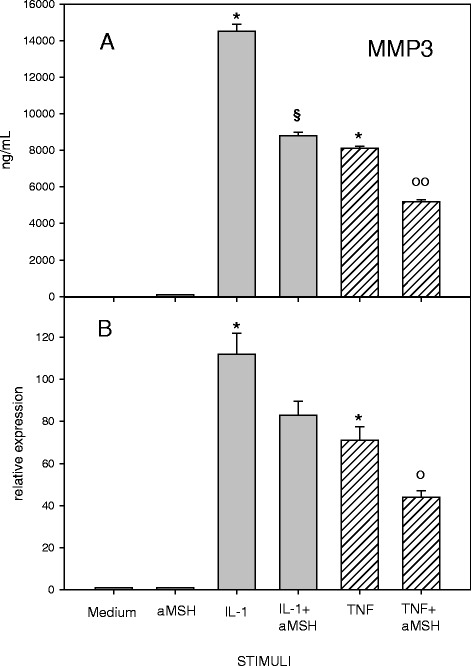
Effect of α-MSH on MMP-3 secretion (**a**) and gene expression (**b**) in IL-1β or TNF-α-stimulated chondrocytes. Human primary chondrocytes were incubated for 4 h at 37 °C with 10^−6^M α-MSH and further cultured for 40 h in the presence or absence of 10 ng/mL IL-1β or TNF-α. MMP-3 concentration in the supernatans (**a**) and gene expression (**b**) were then measured. Data from 4 separate experiments (± S.E.M.) are shown. * *p* < 0.0001 vs Medium; ^**§**^ *p* < 0.001 vs IL-1β; **°** *p* < 0.01 vs TNF-α; **°°** *p* < 0.001 vs TNF-α

**Fig. 4:**
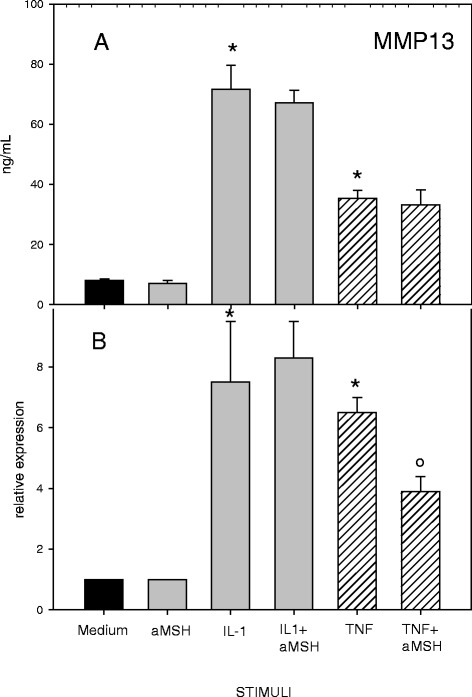
Effect of α-MSH on MMP-13 secretion (**a**) and gene expression (**b**) in IL-1β or TNF-α-stimulated chondrocytes. Human primary chondrocytes were incubated for 4 h at 37 °C with 10^−6^M α-MSH and further cultured for 40 h in the presence or absence of 10 ng/mL IL-1β or TNF-α. MMP-13 concentration in the supernatans (**a**) and gene expression (**b**) were then measured. Data from 4 separate experiments (± S.E.M.) are shown. * *p* < 0.0001 vs Medium; °*p* < 0.05 vs TNF-α

A significant inhibition of MMP-3 gene expression and secretion from IL-1β- or TNF-α-stimulated chondrocytes was induced by α-MSH (Fig. 3). On the other hand, α-MSH did not modify the release of MMP-13 by cytokine-stimulated chondrocyte but the peptide caused a decreased gene expression of the molecule that was significant for TNF-α-stimulated cells (Fig. 4)

**Fig. 5:**
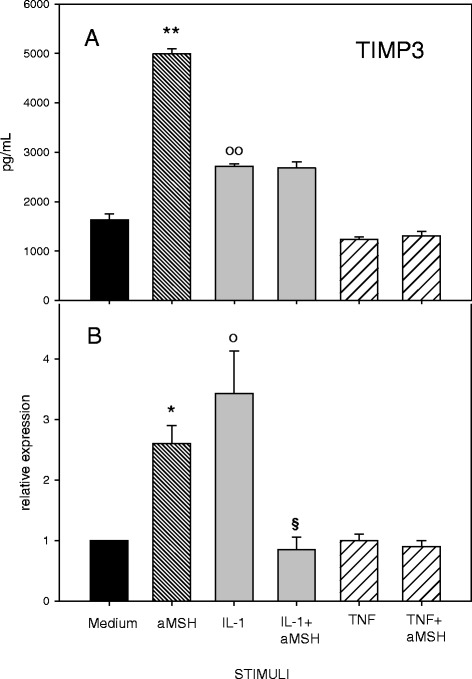
Effect of α-MSH on TIMP-3 secretion (**a**) and gene expression (**b**) in IL-1β or TNF-α-stimulated chondrocytes. Human primary chondrocytes were incubated for 4 h at 37 °C with 10^−6^M α-MSH and further cultured for 40 h in the presence or absence of 10 ng/mL IL-1β or TNF-α. TIMP-3 concentration in the supernatans (**a**) and gene expression (**b**) were then measured. Data from 4 separate experiments (± S.E.M.) are shown. *P < 0.002 vs Medium; **p < 0.001 vs Medium; **°** *p* < 0.02 vs Medium; **°°** *p* < 0.002 vs Medium; ^**§**^ *p* < 0.05 vs IL-1β

**Fig. 6:**
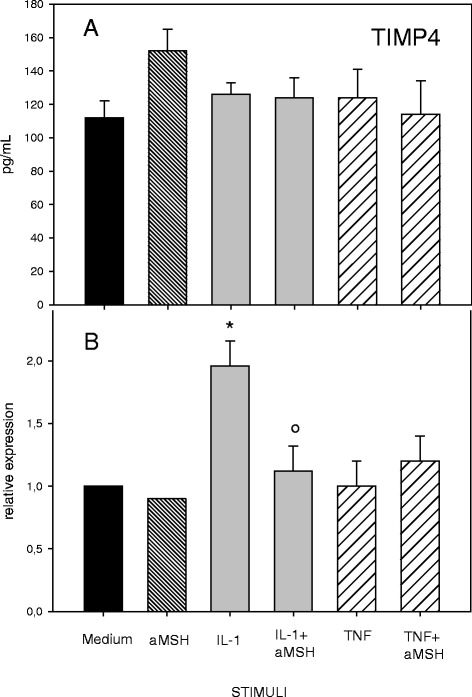
Effect of α-MSH on TIMP-4 secretion (**a**) and gene expression (**b**) in IL-1β or TNF-α-stimulated chondrocytes. Human primary chondrocytes were incubated for 4 h at 37 °C with 10^−6^M α-MSH and further cultured for 40 h in the presence or absence of 10 ng/mL IL-1β or TNF-α. TIMP-4 concentration in the supernatans (**a**) and gene expression (**b**) were then measured. Data from 4 separate experiments (± S.E.M.) are shown. *p < 0.02 vs Medium; **°** *P* < 0.05 vs IL-1β

α-MSH did not modify TIMPs secretion by cytokine-stimulated chondrocyte (Figs. 5 and 6) but the peptide down-regulated the IL-1β-induced gene expression of TIMP-3 (Fig. 5) and TIMP-4 (Fig. 6). Of interest, a significant increase of TIMP-3 gene expression and release was induced by α-MSH (three fold increase) on unstimulated cells (Fig. 5).

**Fig. 7:**
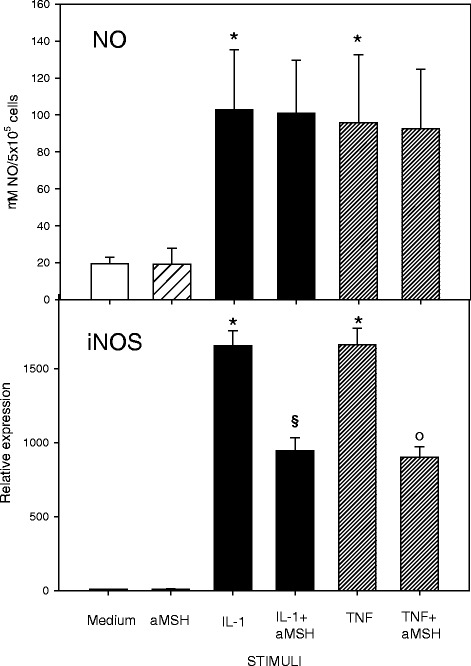
Effect of α-MSH on NO secretion and iNOS gene expression in IL-1β or TNF-α-stimulated chondrocytes. Human primary chondrocytes were incubated for 4 h at 37 °C with 10^−6^M α-MSH and further cultured for 40 h in the presence or absence of 10 ng/mL IL-1β or TNF-α. NO concentration in the supernatans (**a**) and iNOS gene expression (**b**) were then measured. Data from 4 separate experiments (± S.E.M.) are shown. *p < 0.0001 vs Medium; ^§^ *p* < 0.005 vs IL-1β; °*p* < 0.005 vs TNF-α

No inhibition was induced by α-MSH on cytokine-stimulated NO production but the peptide was able to reduce gene expression of iNOS2, the enzyme involved in inducible syntesis of nitric oxide (Fig. 7).

## Discussion

The data show that stimulation of human primary chondrocytes with IL-1β or TNF-α induces an inflammatory phenotype. Both cytokines induced gene expression and secretion of IL-6, IL-8, MMP-3 and MMP-13 associated with a significant production of NO. These results confirm a significant chondrocyte responsiveness to exogenous pro-inflammatory stimuli with amplification of the inflammatory response and possible local cartilage degradation. Under our experimental conditions the anti-inflammatory peptide α-MSH did not modify release or gene expression of IL- 6, IL-8, MMP-13, MMP-3 in resting chondrocytes. These results are not consistent with previous observations by Grässel et al. [10] who, using different chondrocyte culture conditions (micromass pellets) reported an α-MSH-induced down-regulation of IL-1β and TNF-α gene expression and an increased secretion of IL-6 and TNF-α in human chondrocytes. Of interest, we observed that α-MSH was able to induce gene expression and secretion of TIMP-3 in resting cells. These results suggest a potential role for α-MSH in the maintenance of cartilage homeostasis. Indeed the anticatabolic influence of TIMP-3 as an inhibitor of molecules implicated in cartilage degradation, including MMP and aggrecanase-2, is well recognized [19].

When the capacity of α-MSH to modulate IL-1β or TNF-α-induced chondrocyte activation was evaluated, we observed that the peptide did not exert anti-oxidative activity on human chondrocytes as it did not modify cytokine-induced NO production. On the other hand, α-MSH significantly inhibited MMP-3 gene expression and secretion induced by both cytokines further suggesting an anticatabolic role of the peptide. In TNF-α-stimulated chondrocytes the peptide significantly reduced gene expression of IL-6, IL-8, and MMP-13. Conversely, there was no substantial effect in IL-1β-activated cells. These results are consistent with those of Yoon et al. [11] who showed that α-MSH inhibited TNF-α-induced MMP-13 gene expression in a human chondrosarcoma cell line.

The present data are partly consistent with those of Kaneva et al. [13] who reported an inhibitory effect of α-MSH on pro-inflammatory cytokine release (IL-1, IL-6, IL-8) and on MMP-1, MMP-3, MMP-13 gene expression in a TNF-α-stimulated human chondrocyte cell line.

Collectively, these results underscore a potential anti-inflammatory and chondroprotective activity exerted by α-MSH in TNF-α-stimulated human chondrocytes.

In our experimental conditions, the α-MSH-induced inhibition of cytokine-stimulated gene expression of IL-6, IL-8 and MMP-13, and the down-regulation of IL-1β-induced increase in TIMP-3 and TIMP-4 gene expression were not accompanied by a parallel reduction in protein release. A similar discordance between gene expression and protein secretion was previously reported by Grässel et al. [10] using α-MSH-treated micromass pellet cultures.

Although the discrepancy between influences on gene expression and protein release does not have a definite explanation, it may be secondary to an incomplete or late inhibition of gene expression by α − MSH compared to the activation induced by pro-inflammatory cytokines in vitro.

The biological significance of the difference in the inhibitory pattern exerted by α-MSH in TNF-α-stimulated chondrocytes (inhibition of IL-6, IL-8 and MMPs gene expression) relative to IL-1β-stimulated cells (inhibition of TIMP-3 and TIMP-4 gene expression) is likewise unclear. Both IL-1β and TNF-α exert their biological responses mainly through the activation of nuclear factor-κB (NF-κB) signalling pathway and NF-kB suppression is considered the key molecular mechanism of the anti-inflammatory effect of α-MSH [9]. It is possible that different concentrations of the peptide are required to inhibit the activation induced by different cytokines in vitro. An interference exerted by α-MSH on membrane cytokine receptors could be also considered but, while it has been demonstrated that α-MSH potently and selectively reduce membrane binding of IL-1β to T-cell clones, we have no knowledge of similar data for TNF-α.

## Conclusions

The present results indicate that α-MSH exerts chondroprotective activity suggesting a potential role of α-MSH in the maintenance of cartilage homeostasis although the role of the peptide in the control of chondrocyte activation remains to be defined.
